# The inhibition of microRNAs by HIV-1 Tat suppresses beta catenin activity in astrocytes

**DOI:** 10.1186/s12977-016-0256-y

**Published:** 2016-04-08

**Authors:** Luca Sardo, Priyal R. Vakil, Weam Elbezanti, Anas El-Sayed, Zachary Klase

**Affiliations:** Department of Biological Sciences, McNeil Science and Technology Center Room 273, University of the Sciences, 600 S 43rd Street, Philadelphia, PA 19104 USA

**Keywords:** HIV-1, Tat, miRNA, Beta-catenin, Astrocyte, HAND

## Abstract

**Background:**

Long term infection with HIV-1, even in the context of therapy, leads to chronic health problems including an array of neurocognitive dysfunctions. The viral Tat protein has previously been implicated in neuropathogenesis through its effect on astrocytes. Tat has also been shown to inhibit the biogenesis of miRNAs by inhibiting the activity of the cellular Dicer protein in an RNA dependent fashion. Whether there is a mechanistic connection between the ability of HIV-1 Tat to alter miRNAs and its observed effects on cells of the central nervous system has not been well examined.

**Results:**

Here, we examined the ability of HIV-1 Tat to bind to and inhibit the production of over 300 cellular miRNAs. We found that the Tat protein only binds to and inhibits a fraction of the total cellular miRNAs. By mapping the downstream targets of these miRNAs we have determined a possible role for Tat alterations of miRNAs in the development of neuropathogenesis. Specifically, this work points to suppression of miRNAs function as the mechanism for Tat suppression of β-catenin activity.

**Conclusions:**

The discovery that HIV-1 Tat inhibits only a fraction of miRNAs opens new areas of research regarding changes in cellular pathways through suppression of RNA interference. Our initial analysis strongly suggests that these pathways may contribute to HIV-1 disruption of the central nervous system.

**Electronic supplementary material:**

The online version of this article (doi:10.1186/s12977-016-0256-y) contains supplementary material, which is available to authorized users.

## Background

Acquired immunodeficiency syndrome (AIDS) is a complex and multifaceted disease that arises from the infection of CD4+ T-cells by the human immunodeficiency virus (HIV-1). Advances in HIV research have provided us with anti-retroviral drugs that target various viral enzymes like reverse transcriptase, protease and integrase. Although these anti-retroviral drugs control replication of the virus no permanent cure has yet been discovered. Viral replication is positively regulated by the interaction between the viral Tat protein and an RNA element called transactivation response (TAR) element. The Tat protein binds to the nascent TAR RNA and recruits positive elongation complex (pTEFb) that enhances the rate and efficiency of HIV-1 transcription. HIV-1 invades the central nervous system (CNS) within a few weeks of infection and sets the stage for potentially severe inflammatory events. Anti-retroviral drugs poorly penetrate the blood–brain barrier of the CNS allowing low level ongoing replication in the CNS that leads to moderate to severe HIV-associated dementia [1, 2]. The overall spectrum of viral neuropathogenesis is collectively referred to as HIV-associated neurocognitive disorders (HAND). HIV-1 does not infect neurons, implying that HAND must be mediated in an indirect fashion. The HIV-1 Tat protein has been shown to cross the cell membrane and have an effect on bystander cells [3–6]. Furthermore, Tat has been implicated in neurological diseases [7–11] and specific mutations within Tat correlate with clade-specific differences in the prevalence of HAND [11–13]. Specifically, Tat exposure has been shown to contribute to dysfunction of the dopaminergic system, influence dopaminergic uptake kinetics and alter the expression of opiate neuropeptides and receptors [14–16]. Substantial evidence exists that these effects are exacerbated by exposure to opiate drugs [7, 17–19].

Studies have examined the interplay between Tat, infection of astrocytes and opiate drugs that affect brain function. However, none of the studies have examined the role of microRNA (miRNA) in the Tat-HAND axis. RNA interference (RNAi) is a regulatory mechanism found in plants, nematodes, protozoan, Drosophila, and mammalian cells [20–22]. Double stranded RNA is recognized by the RNAi machinery and is processed into small, 21 nucleotide small-interfering RNA (siRNA) which are capable of suppressing gene expression. Endogenously expressed RNA can be involved in RNAi through a pathway involving Drosha mediated cleavage of RNA stem-loops in the nucleus, followed by exportation to the cytoplasm by Exportin-5, and finally cleavage of this pre-miRNA by Dicer to generate a small RNA duplex, called miRNA [23–28]. This miRNA guides the RNA-induced silencing complex (RISC) to a complementary, but not perfectly matching, region in the target mRNA. This association inhibits protein translation with minimal effect on the target mRNA [21, 29, 30].

Preliminary studies have shown that the viral Tat protein inhibits miRNA function [31], not by blocking RISC complex directly, but by binding to the Dicer protein that plays a role in the production of miRNA [32]. A follow up to these studies revealed that alteration of miRNA by Tat–Dicer complex occurs in an RNA-dependent manner [33]. As Tat inhibition of miRNA biogenesis is not due to direct suppression of Dicer, but through the binding of RNA, this opens the intriguing possibility that Tat may be selectively inhibiting specific miRNAs. In this paper, we examine the association of Tat with specific miRNAs and determine what cellular pathways are affected by Tat driven changes in miRNA expression.

Our findings suggest that HIV-1 Tat protein inhibits only a subset of miRNAs and leads to the observed phenotypic changes in astrocytes within the CNS in HIV-infected patients. Our identification of specific miRNAs that are tightly bound to Tat protein suggests downstream effects in Wnt/β-catenin signaling pathway. Wnt/β-catenin signaling is a large and complex pathway known to play a role in numerous cellular activities like cell differentiation, survival and proliferation. Knockdown studies have shown that β-catenin plays a role in repression of basal HIV-LTR transcription [34, 35]. As such, by repressing the rate and efficiency of HIV-1 transcription, β-catenin serves as a cellular restriction factor in the CNS. This work identifies a mechanism by which Tat protein represses β-catenin activity, thereby promoting neuroinflammatory activities in HIV-infected patients.

## Results and discussion

### HIV-1 Tat binds to a specific subset of cellular microRNAs

**Fig. 1:**
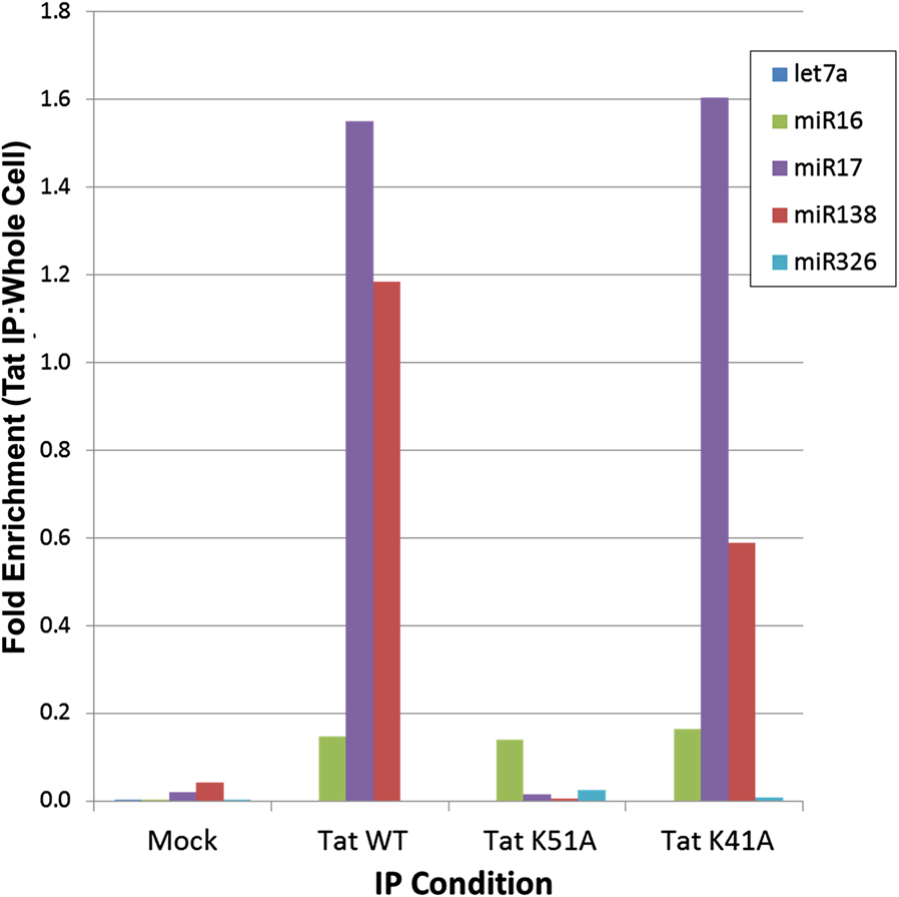
Tat association with cellular miRNA. HEK 293T cells were transfected with Flag Tat-WT, K51A or K41A. Forty-eight hours post-transfection Tat complexes were isolated by anti-Flag IP and RNA was extracted from these complexes or whole cells. RT-qPCR was used to determine if target miRNA associated with Tat complex

Previous studies have shown that Tat interacts with Dicer only in the presence of RNA [33]. Here, we examined which miRNAs are capable of binding to Tat in vivo. In order to identify which miRNAs interact with Tat, we transfected flag-tagged wild-type Tat plasmid into HEK293T mammalian cells and immunoprecipitated using anti-flag antibody, isolated RNA and measured the expression of five miRNAs: let7a, miR16, miR17, miR138 and miR326 (Fig. 1). miR16, miR17 and miR138 showed Tat interaction while let7a and miR326 did not bind to Tat protein. When Tat was mutated at position K51A, a residue previously shown to be critical to Tat inhibition of Dicer activity, the interaction between Tat and miR17 and miR138 was lost [33]. Interestingly, the IP of Tat K41A, which is incapable of transactivating the HIV-1 LTR but still has ability to inhibit Dicer function, showed similar miRNA binding as wild-type Tat.

**Fig. 2:**
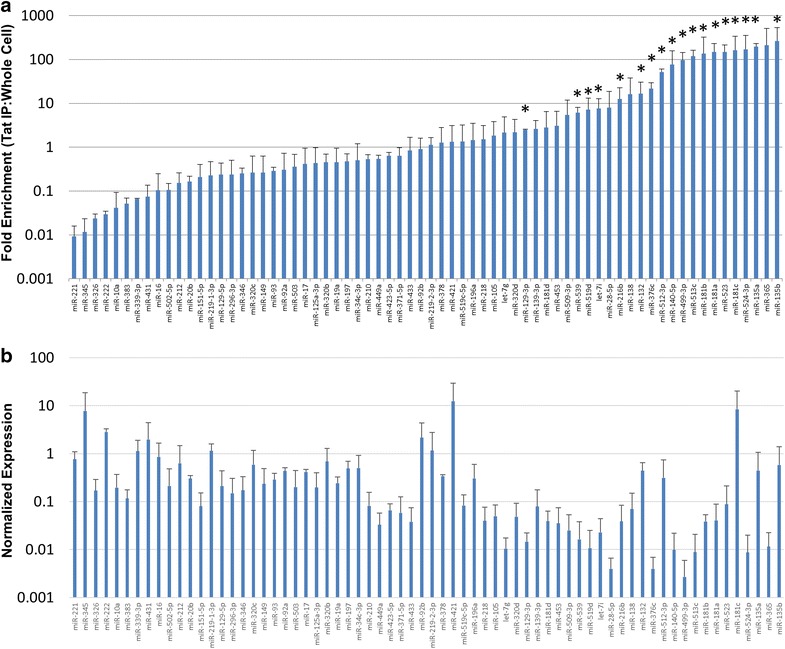
Identification of miRNAs tightly bound by Tat complex. HEK 293T cells were transfected with Flag Tat plasmid and Tat complexes were isolated 48 h post-transfection using anti-Flag IP. **a** RNA from Tat complexes and whole cells was used as input into a polyA mediated RT-qPCR array capable of detecting over 300 human miRNAs. Shown is the fold enrichment in the Tat complex of the 68 miRNAs found to bind to Tat. The miRNAs indicated by *asterisk* show a twofold enrichment in the Tat complex as compared to the whole cell in all replicates. **b** Corresponding levels of miRNA in the whole cell RNA fraction

In order to expand our preliminary findings, we performed immunoprecipitation of flag-tagged wild-type Tat from 293T cells and measured association with 380 human miRNAs (Fig. 2). After making a present-absent call, the data was normalized on a per-array basis and the enrichment of miRNAs by Tat was graphed as the ratio of Tat-associated to the whole cell level of each miRNA. Out of 380 miRNAs measured, 68 miRNAs were found to have at least some interaction with Tat protein (Fig. 2a). Of these 68 miRNAs bound to Tat, 18 miRNAs showed consistent enrichment of greater than twofold and were considered tight Tat binders (asterisk in Fig. 2a). On further analysis on these 18 miRNAs, ten miRNAs were found to be down regulated at the whole cell level when Tat protein was expressed (Table 1). To rule out the possibility that overall expression levels of a given miRNA were influencing the amount of miRNA measured in the Tat IP we graphed the whole cell levels of the 68 Tat associated miRNAs (Fig. 2b). No correlation was found between total expression levels and Tat binding. These results showed that Tat binds and inhibits the production of only a subset of possible miRNAs.

**Table 1:** miRNAs associated with Tat protein

miRNA	Fold enrichment	Down regulated?	β-Catenin	Axonal guidance	Glucocorticoid receptor signaling	Nerve growth factor
let-7i	7.6E+00	Yes	X	X	X	X
miR-129-3p	2.5E+00	Yes	X	X	X	X
miR-132	1.7E+01			X	X	X
miR-135a	2.0E+02	Yes	X	X	X	X
miR-135b	2.6E+02	Yes	X	X	X	X
miR-140-5p	7.6E+01		X	X	X	
miR-181a	1.5E+02	Yes	X	X	X	X
miR-181b	1.4E+02	Yes	X	X	X	X
miR-181c	1.6E+02		X	X	X	X
miR-216b	1.3E+01		X	X	X	X
miR-376c	2.2E+01		X	X	X	X
miR-499-3p	9.6E+01	Yes		X	X	X
miR-512-3p	5.1E+01		X		X	
miR-513c	1.2E+02		X	X	X	
miR-519d	7.2E+00		X	X	X	X
miR-523	1.5E+02	Yes				
miR-524-3p	1.7E+02	Yes		X		
miR-539	6.1E+00	Yes	X	X	X	X

### HIV-1 Tat alters miRNAs with potential roles in the CNS

**Fig. 3:**
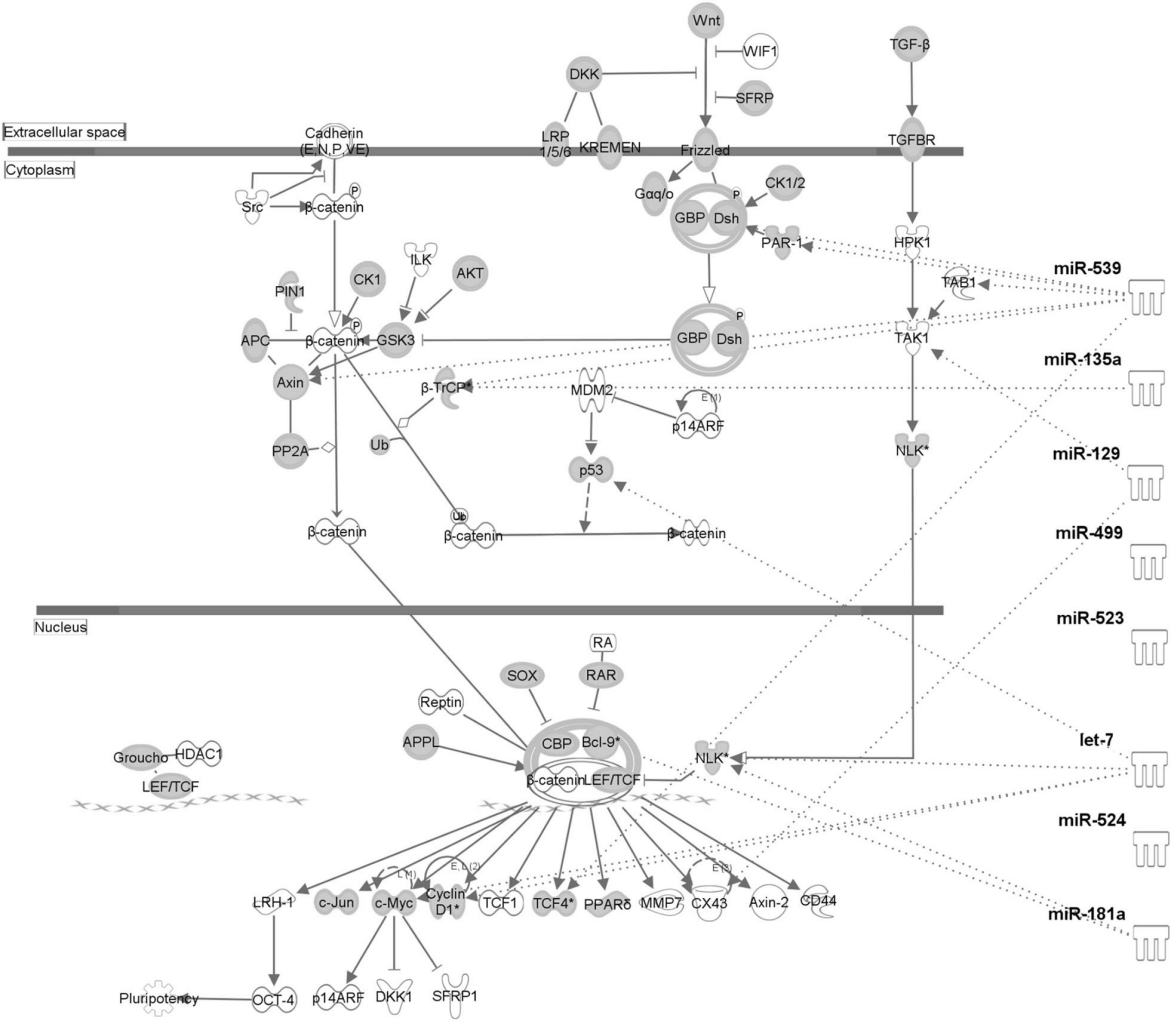
Targeting of the Wnt/β-catenin signaling pathway by Tat altered miRNAs. Ingenuity pathway analysis software was used to predict the downstream targets of the 18 tight Tat binders and to visualize the Wnt/β-catenin pathway. Proteins with symbols filled in *gray* are targets of Tat bound miRNAs. miRNAs confirmed to be down-regulated at the whole cell level are shown on the *right*. The *dashed lines* depict the targets of these eight miRNAs

**Fig. 4:**
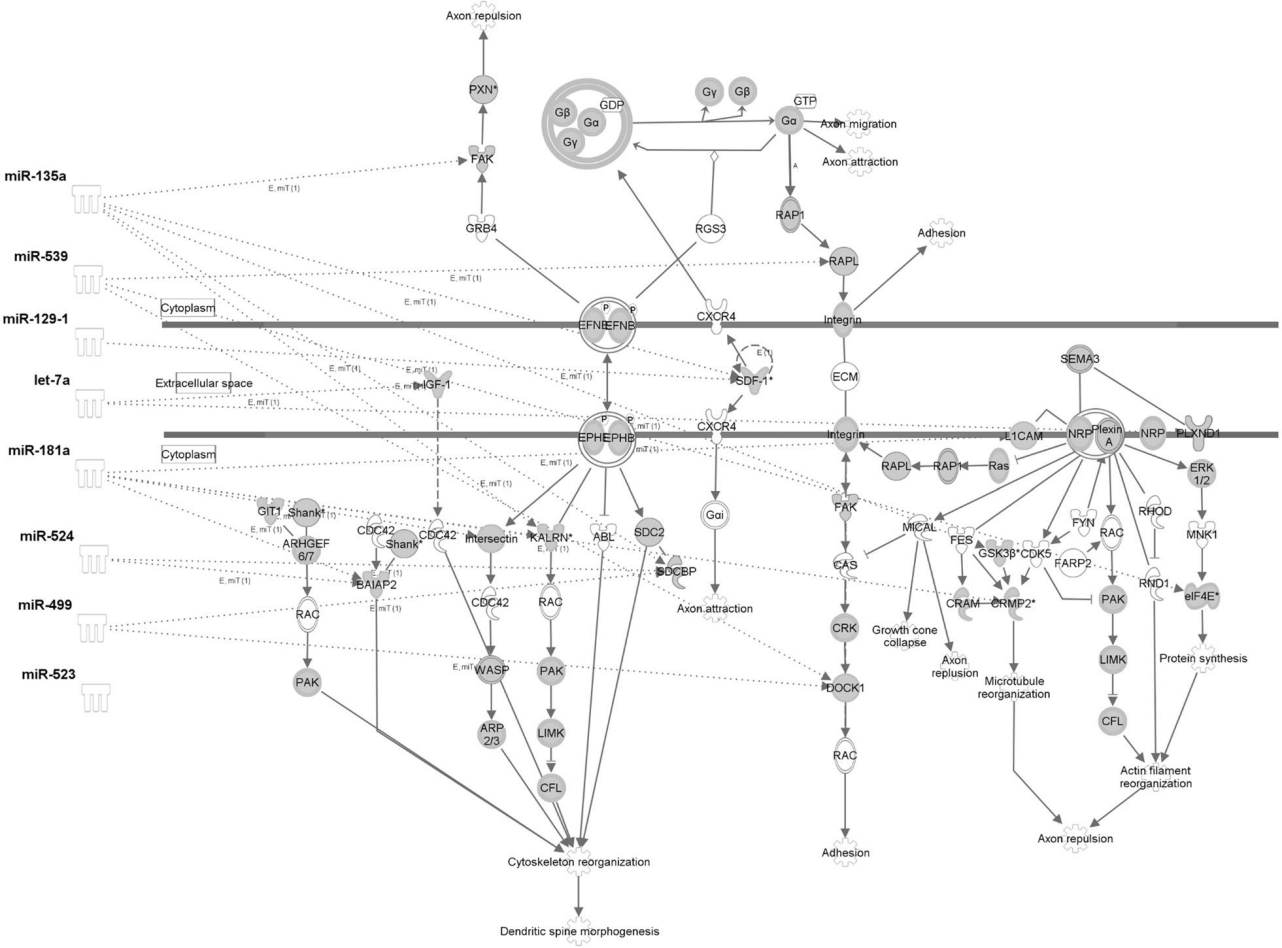
Targeting of the axonal guidance signaling by Tat altered miRNA. Ingenuity pathway analysis software was used to predict the downstream targets of the 18 tight Tat binders and to visualize the Wnt/β-catenin pathway. The targets of the miRNAs bound by Tat are filled in with *gray*. miRNAs confirmed to be down-regulated at the whole cell level are shown on the *left*. The *dashed lines* indicate targets of these eight miRNAs

Each individual miRNA is capable of potentially targeting dozens of mRNA transcripts and, depending on context and the number of targeting miRNA, have wide range of effect on protein expression. As such, we felt it was best to approach the issue of downstream effects in a holistic fashion that would look for the net outcome of changes in all of the 18 tight Tat binders. We employed ingenuity pathway analysis software (IPA) to predict the potential protein targets of the 18 tight Tat binders and then determine what pathways these proteins belong to. IPA analysis showed a strong probability of Tat alteration of miRNAs being involved in several processes related to HIV-1 pathogenesis: Wnt/β-catenin signaling cascade (p = 9.06 × 10^−9^, Fig. 3), axonal guidance (p = 5.92E^−18^, Fig. 4), glucocorticoid receptor signaling (p = 1.23 × 10^−11^, not shown) and nerve growth factor signaling (p = 1.3 × 10^−14^, not shown) [36–40]. Previous studies have shown that alterations of β-catenin in HIV-1 infected CNS is likely a necessary step for infection of astrocytes and eventual neuropathogenesis [38]. From our analysis, we show that Tat has a role in the neuropathogenesis of CNS by its suppression of specific miRNAs that are involved in Wnt/β-catenin signaling or axonal guidance pathway.

### HIV-1 Tat downregulates β-catenin activity in a miRNA dependent manner

**Fig. 5:**
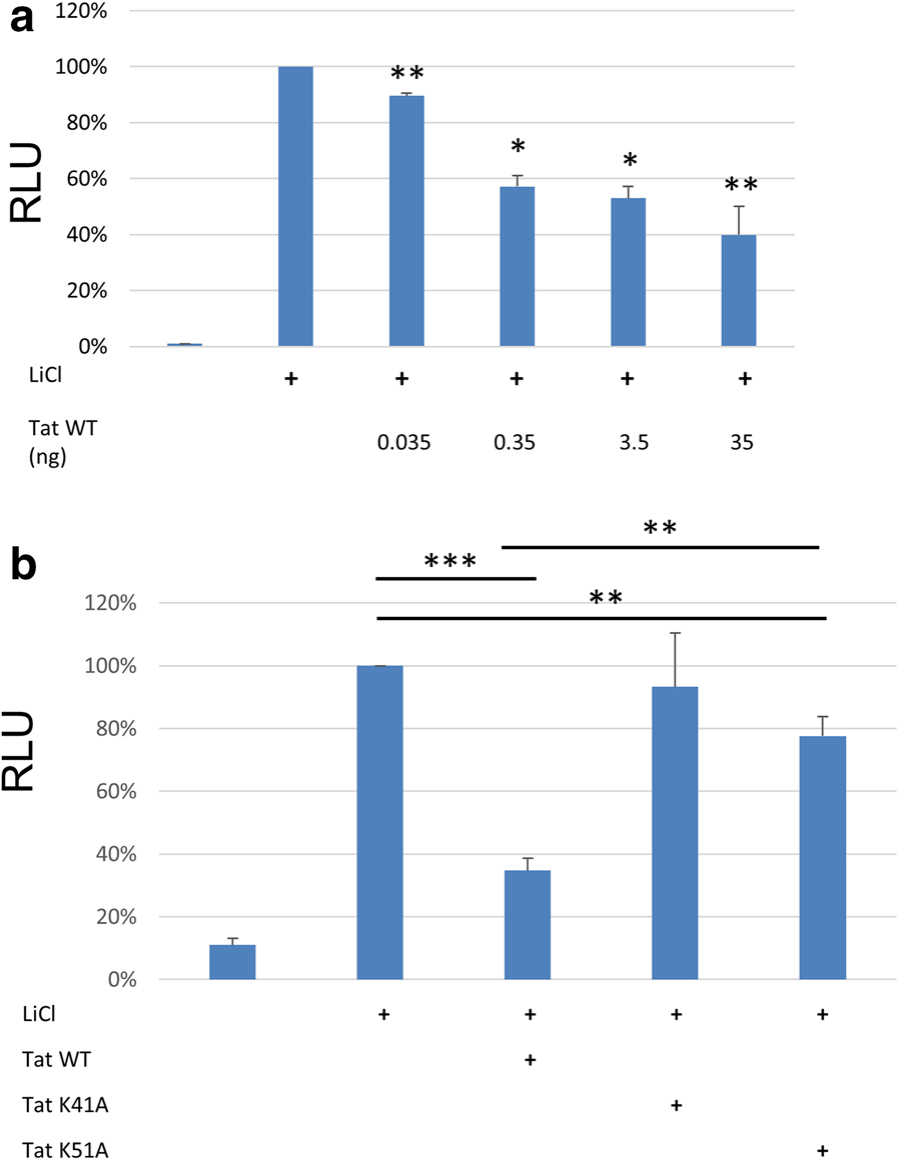
HIV-1 Tat inhibits β-catenin activity in U-87MG cells. **a** U-87MG were transfected with a β-catenin responsive luciferase vector and increasing concentrations of Tat expression vectors at the indicated amounts. Twenty-four hours later the cells were treated with LiCl to induce activation of β-catenin. Twenty-four hours post LiCl treatment luciferase activity was measured and displayed as a percentage of maximal activity. **b** Indicated Tat mutants were transfected into U-87MG alongside reporter and measured as above. *p ≤ 0.05; **p ≤ 0.01; ***p ≤ 0.001

Our IPA analysis suggested an effect on Wnt/β-catenin signaling when wild-type Tat protein is expressed. To confirm an effect on beta-catenin and the involvement of miRNAs, we performed a β-catenin responsive reporter gene assay to look at the β-catenin activity in an astrocyte cell line, U-87MG (Fig. 5). Previous studies have shown that lithium chloride (LiCl) enhances the activity of β-catenin in cells. Therefore, we performed luciferase assay with wild-type Tat in presence of LiCl. Transfection of U-87MG with increasing amounts of wild-type Tat showed a dose dependent decrease in β-catenin activity when compared to just LiCl treatment (Fig. 5a). This is consistent with earlier reports that Tat is capable of inactivating β-catenin. Interestingly, when transfecting Tat K41A a significant decrease in β-catenin activity was not observed (Fig. 5b). A previous study has already identified lysine 41 as an important residue in β-catenin modulation [34]. The Tat K51A mutant, which is incapable of binding to miRNA, induces a slight, but statistically significant suppression of β-catenin activity. However the suppression of β-catenin activity by Tat K51A is significantly weaker than wild-type Tat. This new data confirms a role for lysine 51 and its ability to modulate miRNA interaction and suppression in the ability of Tat to suppress β-catenin activity.

**Fig. 6:**
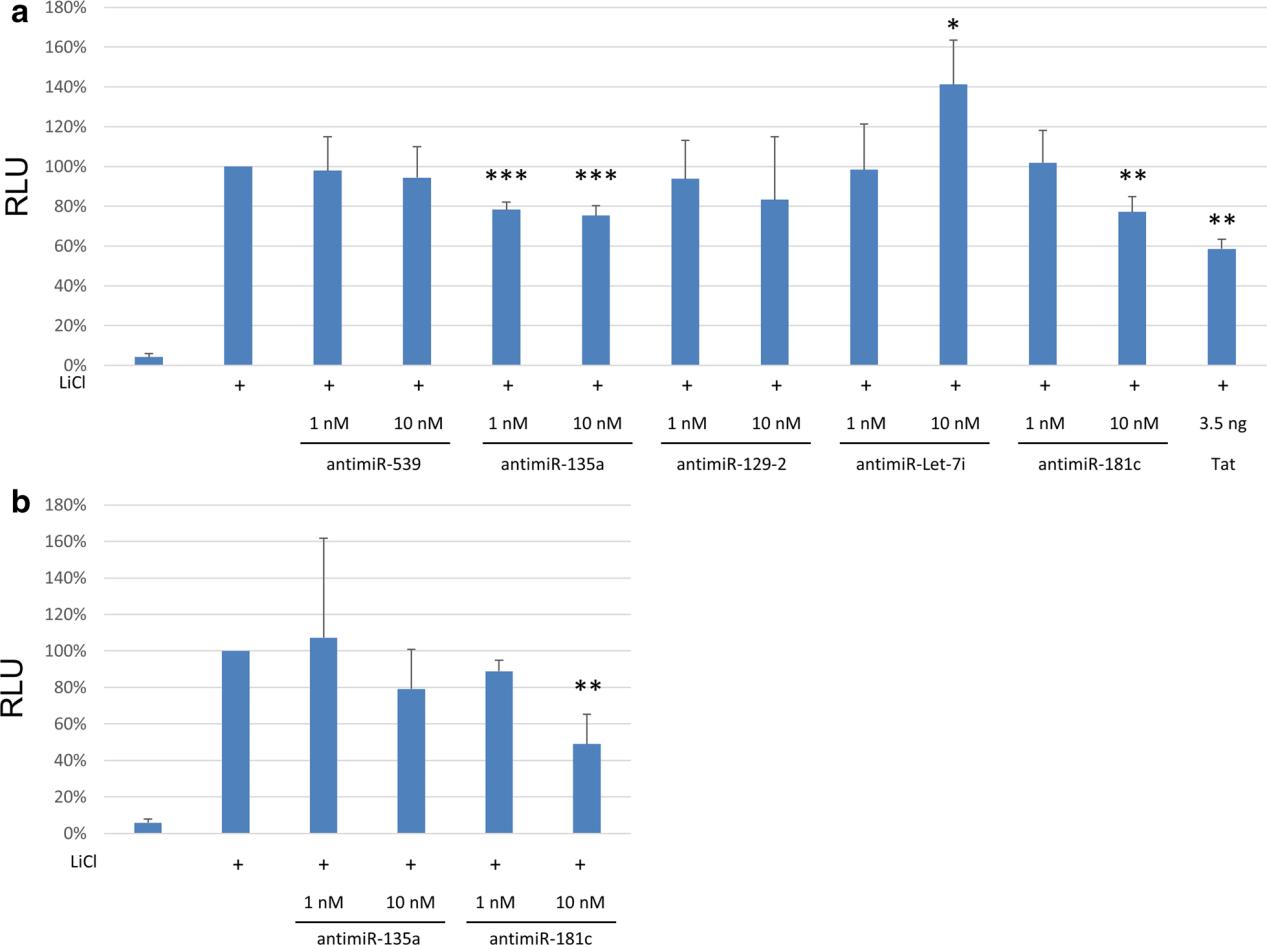
Inhibition of Tat altered miRNAs recapitulates the observed suppression of β-catenin activity in U-87MG and HeLa cells. **a** U-87MG and **b** HeLa cells were transfected with a β-catenin responsive luciferase vector and antagomirs that inhibit the indicated miRNAs. Twenty-four hours later cells were treated with LiCl to induce activation of β-catenin. Twenty-four hours post LiCl treatment luciferase was measured and displayed as a percentage of maximal activity. *p ≤ 0.05; **p ≤ 0.01; ***p ≤ 0.001

To confirm that the identified miRNAs could mediate the downregulation of the β-catenin signaling pathway we used miRNA inhibitors (antagomirs) to block the effect of miRNAs. Antagomirs complementary to miRNAs predicted to target β-catenin were transfected into U-87MG along with a β-catenin responsive reporter gene (Fig. 6a). Inhibition of miR-135 and miR-181 induced a statistically significant reduction of β-catenin activity in U-87MG astrocytes. Blocking the effect of miR-539 and miR-129 had no effect. Interestingly, inhibition of let-7 induced a dose dependent increase in β-catenin activity. The reduction of β-catenin activity mediated by miR-181 was confirmed also in HeLa cells (Fig. 6b).

### HIV-1 Tat alters the morphology of U-87MG astrocyte cells

When U-87MG was treated with LiCl we observed a striking change in morphology that correlated with the activity of β-catenin. Specifically, the cells appeared more spindle-like with significant branching when compared to the normal morphology of U-87MG (Additional file 1: Figure S1). This morphology appears similar to protoplasmic astrocytes seen in brain tissue. However, when wild-type Tat was transfected into U-87MG prior to the introduction of LiCl, the cells seemed to lose their spindle-like structure and appeared smaller, similar to the morphological changes observed in the brains of HIV-1 infected patients suffering from HAND.

## Conclusions

It has been previously reported that HIV-1 Tat protein binds and inhibits Dicer in a RNA-dependent manner. In the current study, we show that Tat protein binds and inhibits the production of only certain cellular miRNAs. We identified 68 miRNAs that have some interaction with Tat protein. Out of these 68 miRNAs, we found that 18 showed more than twofold enrichment with Tat and were categorized as tight Tat binders. When the potential targets of these 18 tight Tat binders were analyzed, it was found that these miRNAs were likely involved in either axonal guidance, glioma signaling, glucocorticoid synthesis or canonical Wnt/β-catenin signaling in the CNS. On further investigating the role of HIV-1 Tat in Wnt/β-catenin signaling via miRNA inhibition, it was found that Tat inhibits the activity of β-catenin in a miRNA dependent manner. From previous studies, it has been noted that repression of β-catenin has a role to play in the neuropathogenesis of HIV. Here, we report that HIV-1 Tat indirectly inhibits the β-catenin activity via miRNA inhibition and this alteration of miRNA likely has a significant role to play in the neuropathogenesis of HAND in HIV-1 infected patients.

Our findings highlight the importance of the lysine residues at both position 41 and 51 of Tat. The K41A mutant and K51A mutant have previously been shown to have different effects: loss of transactivation ability and loss of Dicer inhibition respectively. That both motifs are required for efficient suppression of β-catenin activity is of interest. Additional work must be performed to expand our understanding of the mechanisms by which these motifs inhibit β-catenin. Additionally, other pathways involved in the pathogenesis of HAND that might be affected by alterations in miRNA must be evaluated. Previous studies have shown that opiate drugs like morphine exacerbate the neuropathogenesis in the CNS. This opens the intriguing possibility that opiates and Tat alter similar miRNA subsets. Continued examinations of the alterations of miRNAs, as well as the downstream effects of such alterations, could open up pathways to possible miRNA directed therapies or biomarkers for the diagnosis of HAND.

## Methods

### Cell culture and transfection

HEK 293T and HeLa were cultured in Dulbecco’s Modified Eagle Medium and U-87MG were cultured in Eagles Minimal Essential Media, supplemented with 10 % Fetal bovine serum, 4.5 g/l of l-glutamine and penicillin/streptomycin. Cells were grown in 75 cm^2^ polystyrene flask under standard conditions at 37 °C with 5 % CO_2_. WT-Tat, K41A-Tat, K51A-Tat and CMV-GFP expression vectors were transfected into HEK 293T cells in 9.60 cm^2^ wells with TransFectin Lipid Reagent (Bio-Rad, Hercules, CA, USA). Cells were harvested after 48 h post-transfection, centrifuged for 2 min at 1500 rpm, washed 2 times with ice-cold phosphate buffered saline (PBS), lysed with 50 µl RIPA buffer [(150 mM NaCl, 1 % NP-40, 0.5 % sodium deoxycholate, 0.1 % SDS, 50 mM Tris (pH 8.0)] and put on ice for 30 min. Cell suspensions were centrifuged for 5 min at 14,000 rpm and supernatants were stored at 20 °C for further analysis. For the luciferase assay, U-87MG and HeLa were cultured in 96-well plate and transfected with TransFectin Lipid Reagent (Bio-Rad, Hercules, CA, USA). miRNA inhibitors were obtained from GE Dharmacon (Miridian miRNA inhibitors).

### Co-immunoprecipitation

Co-IP was performed using the Pierce classic IP kit. In brief, 1 mg cell lysate from each transfected sample was prepared with IP lysis buffer. Cell lysate (1 mg) was added to Pierce Spin Column, containing 80 µl of the Control Agarose Resin Slurry and incubated at 4 °C for 1 h on a rocker. The binding of antibody to protein was facilitated by addition of 2 µg of monoclonal anti-Flag antibody (ThermoFisher Scientific, monoclonal clone L5) to tubes containing pre-cleared cell lysate. The tubes were incubated at 4 °C overnight with constant shaking. The prepared Pierce Protein A/G Agarose beads were added to the antibody-protein solution and incubated for 1 h at 4 °C with constant shaking. The antibody-protein-agarose bead solution was centrifuged in a 1.5 ml collection tube (ThermoFisher Scientific, Rockford, IL, USA) at 1000*g* for 1 min. The supernatant was discarded and the pellet was placed in a new collection tube. The pellet was washed three times with Pierce IP Lysis/Wash Buffer and finally with 100 µl of 1× conditioning buffer (diluted from 100× ThermoFisher Scientific Conditioning Buffer). The immune complex (antibody-protein-agarose bead) was eluted by addition of 500 µl of Trizol Reagent (Invitrogen, Carlsbad, CA, USA) to the Pierce spin column.

### RNA extraction and RT-PCR

The homogenized immune complex in Trizol obtained from Co-IP was incubated for 5 min at room temperature and centrifuged at 1000*g* for 1 min to obtain the dissociated nucleoprotein complex in a new collection tube. The elution was followed by addition of 100 µl of chloroform for phase separation as per the manufacturer’s protocol (Invitrogen, Carlsbad, CA, USA). In order to precipitate RNA from aqueous phase, 250 µl of 100 % isopropanol was used. The pellet was washed with 500 µl of 75 % ethanol and centrifuged at 7500*g* for 5 min at 4 °C. Total RNA was resuspended in 40 µl of RNase-free water (5 Prime, Hilden, Germany) and incubated at 60 °C for 15 min. The total yield of isolated RNA was determined using NanoDrop (ThermoFisher Scientifc, Rockford, IL, USA). RNA extraction was repeated in a similar manner with 100 µg of whole cell lysates 89.5 ng of total RNA was used to measure miRNA using the QuantiMiR RT Kit (System Biosciences).

### Real-time qPCR and miRNA profiling

Real-time PCR was carried out using System Biosciences miRNome miRNA Profilers QuantiMir Kit (System Biosciences, Mountain View, CA, USA) following the company protocol. Analysis was limited to the first plate in the microRNome array, which contains the most commonly studied miRNAs. Previous studies have shown that this method allows the detection of both pre-miRNA and mature miRNA [41, 42].

### Luciferase activity

β-Catenin activity was measured by transfection of cells with TCF-LEF Reporter system (SABiosciences). To activated β-catenin, cells were treated with either 10 or 50 mM LiCl. β-catenin responsive luciferase levels were measured using the Dual-Glo^®^ Luciferase Assay System (Promega). Firefly luciferase responsive to beta-catenin was normalized to renilla luciferase internal control.

## Additional file

10.1186/s12977-016-0256-y HIV-1 Tat reverses morphological changes induced by LiCl in U-87MG. U-87MG were transfected with a CMV-GFP vector and Tat expression vector. 24 h later the cells were treated with LiCl to induce activation of β-catenin. Twenty-four hours post LiCl treatment cells were imaged both by bright field (top row) and fluorescence (bottom row) to identify cells that had successfully been transfected with the GFP and Tat vectors.
