# Supplementary figures and images for: The inhibition of microRNAs by HIV-1 Tat suppresses beta catenin activity in astrocytes
Source: Retrovirology. 2016 Apr 8;13:25. doi: 10.1186/s12977-016-0256-y (PMC4826512; doi:10.1186/s12977-016-0256-y)

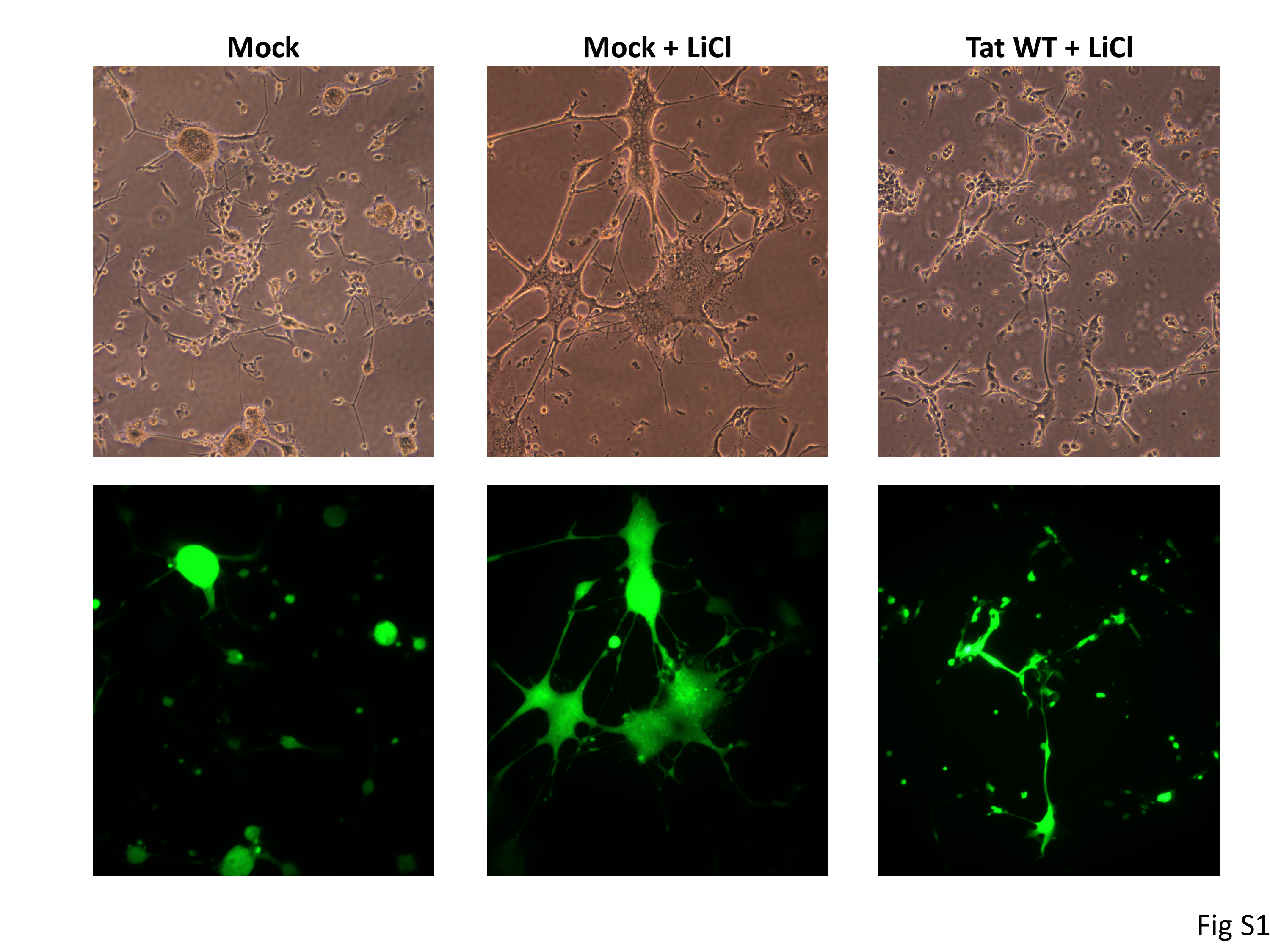

Supplement: Supplementary file 1 — 10.1186/s12977-016-0256-y HIV-1 Tat reverses morphological changes induced by LiCl in U-87MG. U-87MG were transfected with a CMV-GFP vector and Tat expression vector. 24 h later the cells were treated with LiCl to induce activation of β-catenin. Twenty-four hours post LiCl treatment cells were imaged both by bright field (top row) and fluorescence (bottom row) to identify cells that had successfully been transfected with the GFP and Tat vectors. [file 12977_2016_256_MOESM1_ESM.tif]
